# Meniscal allograft transplantation improves patient-reported outcomes in both minimal and moderate knee osteoarthritis at 1 and 2 years postoperatively

**DOI:** 10.1007/s00167-023-07625-3

**Published:** 2023-11-07

**Authors:** Anni Aavikko, Ville Bister, Aleksi Reito, Jan Lindahl

**Affiliations:** 1 Department of Surgery, Päijät-Häme Central Hospital, Lahti, Finland; 2 University of Helsinki, Helsinki, Finland; 3 Department of Surgery, Clinicum, Faculty of Medicine, University of Helsinki, Helsinki, Finland; 4 Department of Orthopaedics and Traumatology, Tampere University Hospital, Tampere, Finland; 5 Department of Orthopaedics and Traumatology, Helsinki University Hospital and University of Helsinki, Helsinki, Finland

**Keywords:** Meniscal allograft transplantation, Knee osteoarthritis, Cartilage damage, Patient-reported outcomes

## Abstract

**Purpose:**

Severe cartilage damage and advanced knee osteoarthritis (OA) might be associated with poor outcomes of meniscal allograft transplantation (MAT). The purpose of this prospective follow-up study was to explore MAT survivorship and patient satisfaction among young patients with symptomatic meniscal deficiency and radiological OA of different Kellgren–Lawrence (K–L) grades.

**Methods:**

Thirty-five consecutive MAT patients were prospectively followed up for 2 years. The lateral meniscus was replaced in 29 patients and the medial meniscus in 6 patients. Outcomes were assessed using the KOOS_4_ composite score, KOOS subscales, Lysholm knee score, and OA K–L grade progression from weight-bearing knee radiographs. For the outcome analysis, patients were categorized into two groups: 19 in Group A (K–L classification 0–1) and 16 in Group B (K–L classification 2).

**Results:**

In terms of KOOS_4_ and Lysholm scores, the patients showed a clinically significant improvement from baseline to the 1-year follow-up (22.2 points, 95% CI 16.6–27.8 for KOOS_4_ and 16.8 points, 95% CI 8.9–24.6 for Lysholm), and the improvement remained at 2 years (20.6 points, 95% CI 13.2–28.1 for KOOS_4_ and 21.5, 95% CI 12.5–30.7 for Lysholm). At the 6-month follow-up, this improvement was not yet observed. Minor between-group differences were observed in the KOOS_4_ and Lysholm scores for the K–L 0–1 and K–L 2 OA groups, but the estimates were imprecise with wide confidence intervals. A clinically relevant difference between these two study groups could not be found at any timepoint. The reoperation rate was higher in the K–L 2 group than in the K–L 0–1 group (31% vs. 11%).

**Conclusions:**

MAT yielded improved patient-reported outcomes and subjective satisfaction at 1 and 2 years postoperatively. The differences from baseline exceeded the minimal clinically important difference (MCID) at all timepoints. The severity of cartilage damage and knee OA in terms of the K–L grade at the time of surgery did not affect the KOOS and Lysholm scores after the MAT procedure. Knee OA progression in terms of K–L grade worsening was not observed in any patients.

**Level of evidence:**

III.

## Introduction

Understanding of the importance of the meniscus for knee joint biomechanics and biology has changed during the last 5 decades [8, 27]. Until the 1980s, arthroscopic partial meniscectomy (APM) was an accepted surgical procedure to treat meniscal tears in a painful knee joint. Currently, APM should be carefully considered for the treatment of knee pain, since studies have shown that while APM may offer short-term pain relief in the long term, it is associated with an increased risk of progression of knee osteoarthritis (OA) [25, 26].

Traumatic meniscal tears are thought to occur due to excessive forces in an otherwise healthy knee. Discoid menisci have a higher rate of tears requiring resection [3]. Today, the first-line treatment for traumatic vertical longitudinal and deep radial meniscal tears is repair whenever possible [1]. However, there is still a group of patients whose meniscal tears are complex and difficult to repair or whose previous surgical repairs have failed. For this group, meniscal allograft transplantation (MAT) may be the treatment of choice. The first human MAT operation was performed in the 1980s [27]. Long-term outcome studies have shown that MAT seems to improve pain and function in young patients with symptomatic meniscal deficiency [7, 10, 21, 28].

Candidates for MAT are young, healthy, and active patients who are symptomatic after undergoing meniscectomy and have not had satisfactory results with further nonoperative management. Evidence of severe knee OA with deep cartilage damage has generally been reported by most publications as a contraindication for MAT grafts, as graft survivorship may be compromised [5]. In most publications, radiographic OA Kellgren–Lawrence (K–L) ≥ 3 has been considered a contraindication for MAT [27].

According to the International Meniscus Reconstruction Forum statement regarding a selected group of younger arthritic patients in whom operative treatments have failed and no other surgical option exists, MAT can be thought of as a bridging solution. In addition, MAT can be performed as a concomitant procedure to correct alignment with distal femoral osteotomy (DFO) or high tibial osteotomy (HTO) and/or articular cartilage repair (ACR) in a meniscus-deficient compartment [10, 40]. Currently, the most discussed issue is whether MAT can provide long-term prevention or delay of articular cartilage degeneration and OA [20, 28].

The purpose of the prospective follow-up study was exploring of MAT survivorship and patient satisfaction among a sample of young patients with symptomatic meniscal deficiency, cartilage damage, and radiological OA of different K–L grades. In addition, the trends of progression of knee OA among young MAT patients during follow-up were evaluated.

## Materials and methods

Approval for the study (HUS/234/2020) was obtained from the Research Board of the Department of Musculoskeletal and Plastic Surgery, Helsinki University Hospital, Helsinki, Finland.

### Study subjects

Orthopaedic surgeons identified potential patients for MAT among those referred to the outpatient clinic. Patients eligible for the study were those with disabling unicompartmental knee pain after a (sub)total meniscectomy and with knee OA K–L 0–2. The patients were invited to an outpatient clinic visit, and they were diagnosed according to clinical and radiologic examinations, including MRI and weight-bearing knee and mechanical axis radiographs. Exclusion criteria were patients older than 45 years, body mass index (BMI, calculated weight in kilograms divided by the square of the height in metres) more than 40, and poor cooperation (drug or alcohol abuse or inability to follow the postoperative rehabilitation protocol). All patients had undergone several knee operations before the current operation. Patients were informed about the prospective follow-up protocol.

### Baseline data

The OA K–L grade was diagnosed from weight-bearing radiographs of the affected knee. The severity of cartilage degeneration was determined from knee MRI and during arthroscopy using the International Cartilage Repair Society (ICRS) score [23]: grade 0, normal; grade 1, nearly normal (superficial softening and/or superficial fissures and cracks); grade 2, abnormal (lesions extending to < 50% of the cartilage depth); grade 3, severely abnormal (cartilage defects extending to > 50% of the cartilage depth and down to the subchondral bone); and grade 4, severely abnormal (complete defect). The mechanical axis was measured from weight-bearing mechanical axis radiographs.

### Surgical technique

The arthroscopically assisted MAT technique was used without a tourniquet under general anaesthesia. Routine diagnostic arthroscopy and cartilage injury evaluation (ICRS) were first performed, followed by the MAT procedure. Meniscal allografts were frozen and nonirradiated from the Helsinki University Hospital Bone and Soft Tissue Bank. Initially, soft tissue meniscal allografts (11 patients) were used, and double bone plug meniscal allografts were used later.

The bone plugs were prepared to fit a diameter of 8 mm and a length of 10 mm. Meniscal allografts were fixed with loop sutures (#2FiberLoop®, Arthrex, Naples, Florida, USA) to the horns. Tunnels were drilled from the anteromedial or anterolateral tibia and the horn sockets with the retro drill technique. The loop sutures were passed through the tunnels and tied over suture buttons in the tibia. The allografts were also fixed to the capsule with vertical inside–out and all-inside sutures. Valgus deformities of the distal femur (≥ 3°) were corrected with lateral opening wedge distal femoral osteotomy (DFO), and tibial varus (≥ 3°) was corrected with a medial opening wedge high tibial osteotomy (HTO). Local deep cartilage lesions (ICRS grade 4) were treated with microfracture if it was deemed clinically relevant during the first half of the study period. In addition, failed anterior cruciate ligament (ACL) reconstructions were treated simultaneously with ACL revision reconstruction. The operations were performed by the last author.

Postoperatively, rehabilitation included a hinged knee brace, crutches, and partial weight-bearing for 6–8 weeks. Toe touch weight-bearing was allowed for the first 4 weeks, followed by 2–4 weeks of half weight-bearing. Knee flexion was limited to 60° for the first 4 weeks and to 90° for the next 2 weeks. Full range of motion (ROM) was allowed after 6 weeks. Cycling exercises began after 8 weeks. Running was allowed after 6 months.

### Follow-up protocol

Patients were seen at follow-up visits conducted at 3 and 6 months and at 1 and 2 years after the operation. Weight-bearing knee radiographs were taken at these timepoints. MRI examination was conducted when deemed clinically relevant. At the follow-up visit, an interview concerning symptoms and a clinical examination were performed by the operating surgeon. Patient-reported outcome measures (PROMs) were collected before the surgery, at 6 and 12 months, and thereafter once a year.

### Outcomes

The outcomes of this study consisted of objective and patient-reported outcomes.

#### Primary outcome measure

The Knee Injury and Osteoarthritis Outcome Score, KOOS_4_ composite score was used as the primary outcome measure [29]. For the analysis, the composite KOOS_4_ score was calculated with a mean score for symptoms, pain, sport and quality of life subscales for each patient [9]. Scores were presented as 0–100, with 100 being the best possible score. A minimal clinically important difference (MCID) of 10 points was used [2].

#### Secondary outcome measures

Secondary outcome measures were divided into radiographic (K–L and OA progression), patient-reported outcomes (KOOS subscales, Lysholm knee score), subjective satisfaction, and adverse events (need for revision surgery, complications). OA progression was monitored at the follow-up visits using weight-bearing radiographs of the knee. For the Lysholm score, a minimal clinically important difference (MCID) of 4.2 points was used [25].

Data on adverse surgical complications (deep wound infection, deep venous thrombosis, meniscal allograft failure, neurological complication) and minor complications (superficial wound infection, pain) were collected. Meniscal allograft failure was defined as the removal of the complete allograft or a revision MAT procedure.

### Study groups

The study population comprised 35 consecutive patients (20 women, 15 men). The median age of the patients was 27 years (range 17–43 years). The first patient was 43 years; all others were below 40 years of age. The baseline characteristics of the study population are given in Table 1. MAT surgeries were performed between 2011 and 2021.

**Table 1:** Baseline characteristics of MAT population (*N* = 35)

	K–L Group A (*N* = 19)	K–L Group B (*N* = 16)
Gender female/male—*n* (%)	11 (57.8%)/8 (42.1%)	9 (56.2%)/7 (43.8%)
Age (years)—median (range)	27 (19 –43)	27 (17–43)
Lateral compartment/medial compartment—*n* (%)	16 (84.2%)/3 (15.8%)	13 (81.3%)/3 (18.8%)
Etiology; post traumatic/discoid meniscus/other—*n* (%)	13 (68.4%)/5 (26.3%)/1 (5.3%)	15 (93.8%)/1 (6.3%)/0
No. of previous surgeries—median (range)	3 (0 –5)	3 (1 –5)

For the outcome analysis, patients were divided into two groups according to the degree of OA in preoperative weight-bearing knee radiographs: Group A K–L classification 0–1 (19 patients) and Group B K–L classification 2 (16 patients). The ICRS scores of cartilage damage in Group B were greater than those in Group A, such that grade 4 cartilage damage was seen in 62% of patients in Group B and in 26% of patients in Group A (Table 2). The main follow-up point was at 2 years.

**Table 2:** Grade of cartilage damage related to the OA K–L grade in MAT patients (*N* = 35)

Osteoarthritis K–L group (grade)	Cartilage damage ICRS grade (maximum)				Total patients
	1	2	3	4	
Group A (0–1)	0	5 (26%)	9 (48%)	5 (26%)	19
Group B (2)	0	2 (13%)	4 (25%)	10 (62%)	16

Prior to the MAT procedure, 1–5 knee operations were performed (median: 3 operations/patient). The aetiology of meniscal disease was trauma for 26 participants, discoid meniscus for 6 participants, and other causes for 3 participants. Eight of the patients had a combined ACL and meniscus rupture (23%). A total of 33 patients (94.3%) answered the PROM questionnaires at least once.

### Statistical analysis

Continuous variables are herein described using means and the associated confidence intervals. Parametric tests were used to examine within- and between-group differences. A paired *t* test was applied to investigate differences between baseline and the follow-up point. A linear mixed model was used to investigate differences between patients with K–L 0–1 and K–L 2 OA. Repeated measures from baseline and follow-up timepoints were used as the outcome, and the group difference was interpreted as the interaction between the K–L group and the timepoint. The given patient was included as a random factor. RStudio v2021.09.1 (Posit PBC, USA) was used for the analysis.

## Results

The numbers of lateral (LMAT) and medial meniscus transplants (MMAT) were 29 and 6, respectively. No bilateral transplantations were performed. As a concomitant procedure to MAT, distal femoral osteotomy (DFO) was performed for 6 patients (17%), high tibial osteotomy (HTO) for one patient (3%), revision ACL surgery for 6 patients (17%), and microfracture of local ICRS grade 4 cartilage lesions for 8 patients (3, tibia condyle; 2, femur condyle; and 3, trochlea). In five of these patients (14%) microfracturing was the only concomitant procedure, while the three others underwent both DFO and microfracturing.

### Patient-reported outcomes

**Fig. 1:**
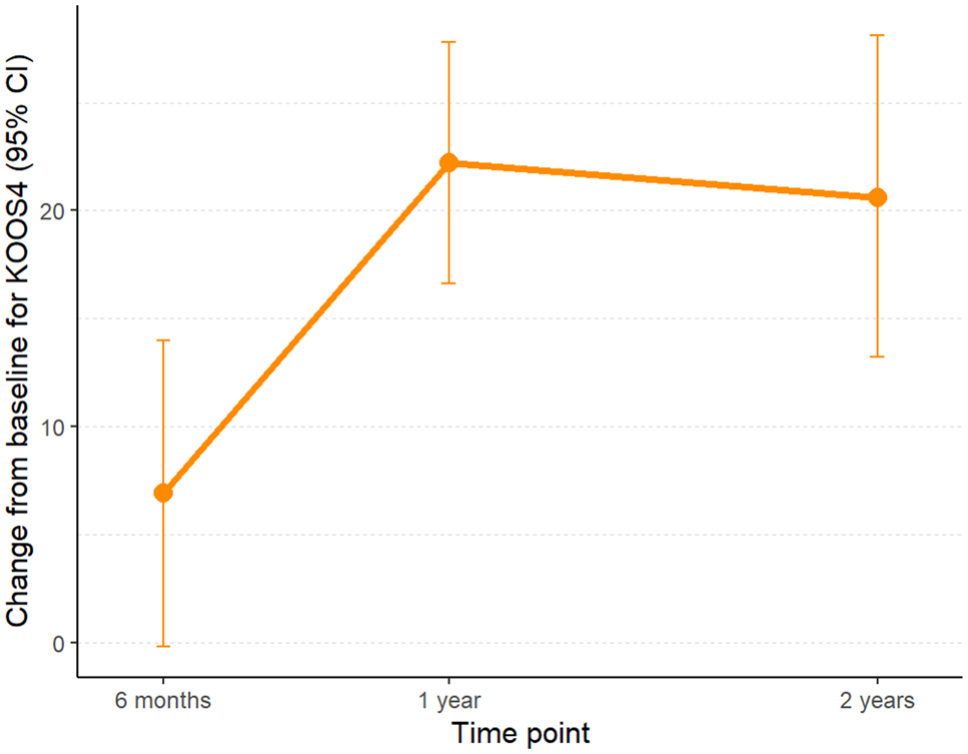
Mean change of KOOS_4_ scores from baseline to follow-up at 2 years in all MAT patients

**Fig. 2:**
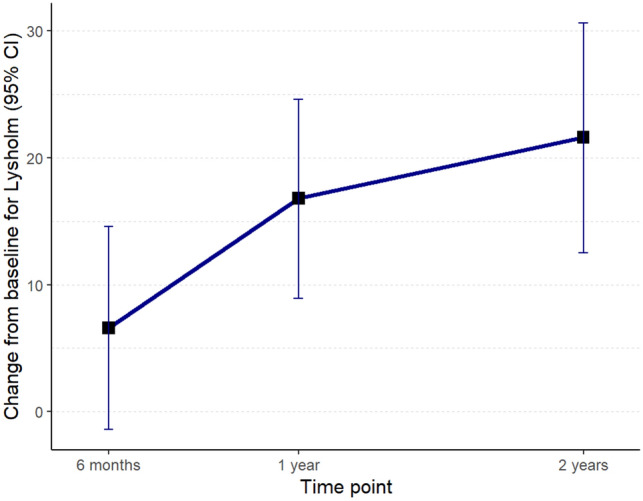
Mean change of Lysholm scores from baseline to follow-up at 2 years in all MAT patients

In terms of KOOS_4_ and Lysholm scores, the patients had a clinically significant improvement from baseline to the 1-year follow-up; the improvement remained at 2 years (Figs. 1 and 2). However, at the 6-month follow-up, this improvement was not yet observed. Similar findings were seen in the KOOS_4_ subdomains (Table 3).

**Table 3:** Mean and SD values in KOOS subgroups among both study groups

	Symptoms		Pain		ADL		Sport		QOL	
	K–L(A)	K–L(B)	K–L(A)	K–L(B)	K–L(A)	K–L(B)	K–L(A)	K–L(B)	K–L(A)	K–L(B)
Preop (SD)	51.8 (16.1)	56.6 (13.)	73.6 (19.3)	66.9 (22.0)	86.6 (12.5)	78.9 (22.0)	50.9 (28.2)	37.7 (29.8)	43.0 (21.0)	31.8 (21.9)
1 year postop	76.6 (10.7)	64.1 (23.5)	83.7 (11.5)	84.7 (17.7)	83.4 (12.9)	92.0 (12.9)	64.2 (19.7)	74.0 (21.5)	66.3 (11.6)	68.8 (19.5)
2 years	74.7 (16.6)	82.9 (16.1)	81.2 (17.5)	89.5 (8.8)	90.1 (10.6)	96.2 (4.2)	60.4 (21.7)	77.2 (16.2)	58.0 (22.7)	66.0 (16.9)

**Fig. 3:**
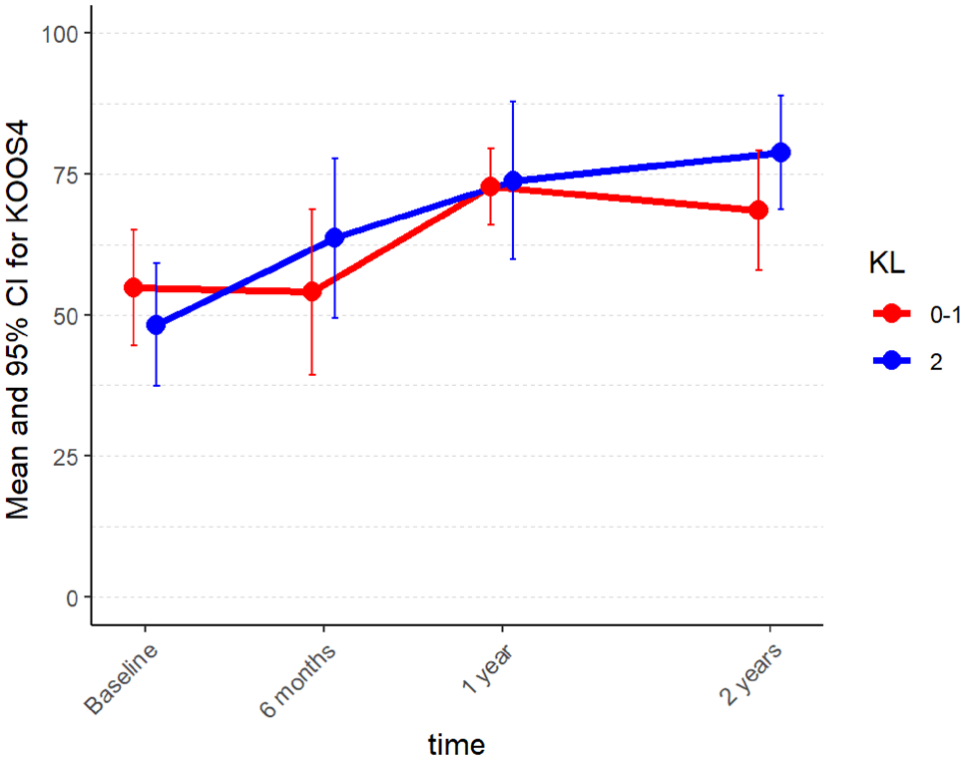
Mean KOOS_4_ scores in Group A (K–L 0–1) and Group B (K–L 2) from baseline to follow-up at 2 years

**Fig. 4:**
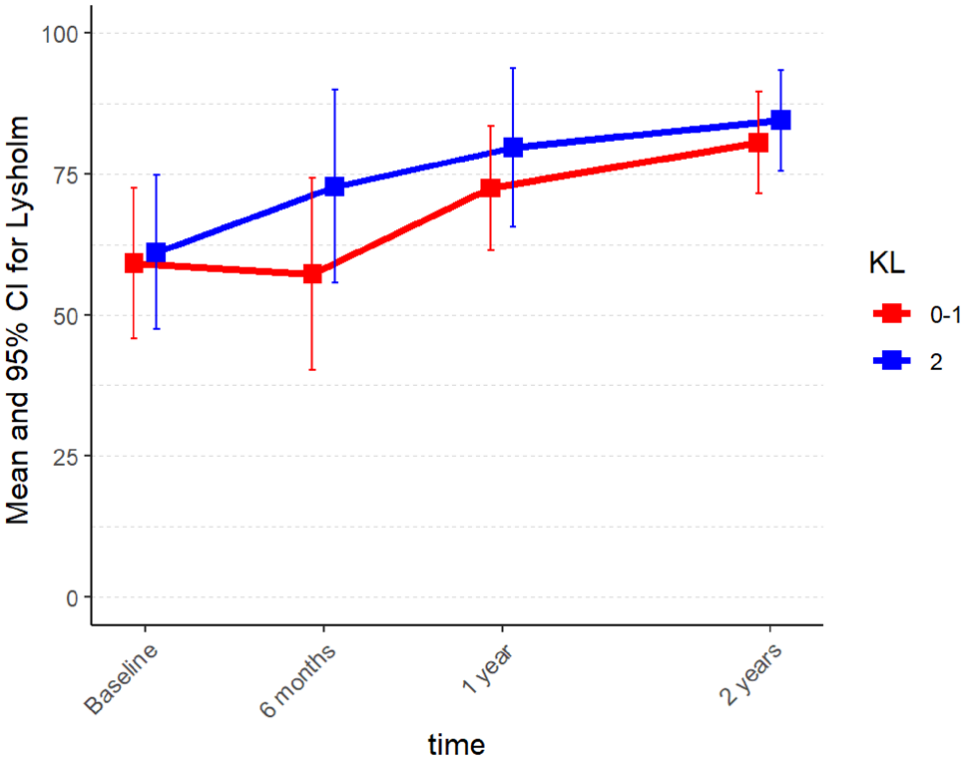
Mean Lysholm scores in Group A (K–L 0–1) and Group B (K–L 2) from baseline to follow-up at 2 years

Minor between-group differences in the KOOS_4_ and Lysholm scores were observed for K–L Group A and K–L Group B, but the estimates were imprecise with wide confidence intervals (Figs. 3 and 4). A clinically relevant between-group difference could not be found at any timepoint (Table 4).

**Table 4:** Mean and SD values of KOOS_4_ and Lysholm scores in all patients and between K–L groups A and B (within group difference)

	KOOS4	Lysholm	KOOS4	Lysholm
	Within group change for baseline all patients	Within	Between group difference for KL	Between
Baseline			6.1 (− 7.0 to 19.2)	− 2.4 (− 18.1 to 13.3)
6 months	6.9 (− 0.19 to 14.0)	6.6 (− 1.4 to 14.6)	− 3.8 (− 18.7 to 11.1)	− 8.3 (− 25.8 to 9.3)
1 year	22.2 (16.6 to 27.8)	16.8 (8.9 to 24.6)	4.3 (− 10.1 to 18.7)	− 2.5 (− 19.4 to 14.3)
2 years	20.6 (13.2 to 28.1)	21.6 (12.5 to 30.6)	− 1.7 (− 16.4 to 12.9)	5.2 (− 12.0 to 22.4)

### Osteoarthritis progression

Between the preoperative assessment and the final follow-up at 2 years, the K–L grade assessed in weight-bearing radiographs did not deteriorate in any patient.

### Complications/adverse effects

No serious adverse effects or postoperative infections were observed during the follow-up. Reoperations were conducted for 7 patients (20%). Five patients had MAT tears either of the body or at root fixation requiring repair (*n* = 4) or partial resection (*n* = 1). Two patients had limitations of knee motion requiring debridement arthroscopy. Reoperations were performed for two patients in K–L Group A (11% of patients in Group A) and five patients in K–L Group B (31% of patients in Group B).

## Discussion

The most important finding of the present study was that MAT increased patient satisfaction and patient-related outcome measures in both K–L grades 0–1 and K–L grade 2 knee OA groups in a 2-year follow-up. Patients in both study groups had clinically significant improvement in KOOS_4_ and Lysholm knee scores from baseline to the 1-year follow-up, and the improvement remained at 2 years. In addition, knee OA progression in terms of K–L grade worsening was not observed in any patients.

Since the 1980s, many studies have been published concerning MAT survivorship and patient satisfaction after the procedure. Equally improved clinical outcomes have been generally reported after MMAT and LMAT [15, 32, 37]. A recent systematic review found no significant risk for graft failure between patients receiving either MMAT or LMAT [17]. In most studies, MAT seems to decrease pain in the affected compartment and provides subjective improvement in up to 70% of patients [11, 30, 38]. Patient satisfaction seems to be also high at long-term follow-up, though revision and conversion to arthroplasty rates increase by time [35].

Significant improvements in PROMs (KOOS and Lysholm scores) have also been reported [13, 31, 34, 36, 39] which is similar to our results, as clinically significant increases were seen in the KOOS_4_ score, most KOOS_4_ subscale scores, and the Lysholm score at the 2-year follow-up.

Only a few studies have reported changes in the OA K–L score after MAT. In one study a worsening of the K–L classification by one point was seen in 14 of 33 patients (42%) in a mean 8.8-year follow-up [33] and in another study with a shorter follow-up (mean 2.6 years), a worsening of knee OA was seen in 8 of 36 patients (22%) [12]. A study conducted with young athletes with a mean follow-up of 3.3 years reported that worsening of the K–L stage by one or more points was seen in 5 of 10 patients (50%) [6]. No worsening of knee osteoarthritis according to the K–L classification was observed in our study at the 2-year follow-up.

A systematic review by Harris et al. [14] found that clinical outcomes were similar between those who underwent MAT with or without concomitant cartilage repair/restoration procedures. Another systematic review and meta-analysis stated that there seemed to be no significant difference between the postoperative PROMs in terms of isolated MAT and MAT combined with other procedures, including cartilage procedures. They could not draw conclusions about the differences in complication, reoperation, survivorship, and failure rates between the two groups because of insufficient data [18]. In our study, microfractures were performed on eight patients with local ICRS grade IV cartilage damage as an associated procedure, but no other cartilage procedures were conducted despite the preoperative cartilage state. According to current knowledge, cartilage procedures used in combination with MAT may not be clinically effective [14, 18].

It has been questioned whether MAT is a meaningful procedure when osteotomy is performed on a malaligned knee with advanced chondral damage and knee OA or when ACL reconstruction is performed on an unstable knee. Bloch et al. assessed the impact of concomitant operations and the influence of articular cartilage lesions on outcomes [4]. They found that the addition of osteotomy or ACL reconstruction led to results similar to those in patients with MAT as the only procedure conducted on normally aligned and stable knees. Kempshall et al. [16] evaluated the concomitant use of osteotomy on patient-reported outcomes with multivariate analysis. They found a negative association between concomitant osteotomy and changes in delta KOOS, Lysholm and IKDC scores, supporting the theory that osteotomy alone is not responsible for patients’ improvement in symptoms. A recent study by Lee et al. clarified the biomechanical effects of the realignment procedure with LMAT [20]. Their results showed that isolated distal femoral osteotomy was inadequate to restore load distribution in meniscus-deficient knees, while concomitant LMAT restored near normal forces and improved the lateral compartment biomechanical profile. Conversely, it is unlikely to achieve as good results with MAT surgery alone as with concomitant corrective osteotomy in the presence of malaligment of the lower extremity, and concomitant ACL reconstruction in the presence of knee instability. In our study the outcomes of isolated MAT and combined MAT and osteotomy (20%), and MAT and ACL reconstruction (17%) were evaluated as a whole, which limits the interpretation of the results. More studies with similar outcome measures are required specially to verify the effect of osteotomy on the clinical outcomes of MAT.

The evidence whether MAT procedures are chondroprotective has been scarce. A recent quantitative 3-T magnetic resonance imaging T2 mapping study by Lee et al. found that lateral MAT without cartilage procedures seems to have a chondroprotective effect on the weight-bearing cartilage [19]. In their study patients with full-thickness cartilage defects were excluded. In addition, patients who underwent concomitant cartilage repair procedures, realignment osteotomies or ligament reconstruction were excluded to be able to investigate the chondroprotective effect of isolated LMAT. Future short- and mid-term advanced imaging studies with quantitative MRI may improve noninvasive evaluation of the chondroprotective effect of MAT on the weight-bearing cartilage surfaces.

Interestingly, MCID was reached between the preoperative state and the 2-year follow-up more often among patients in K–L Group B than among those in K–L Group A, as assessed on the KOOS_4_ subgroup scales. On the pain subscale, this can be explained by decreased load to the cartilage after the procedure. The greatest increase in subscale scores was seen in sports, where the increase between the preoperative state and the 2-year follow-up was 39.6 points (Table 3). A retrospective case series of young high-level athletes experiencing postmeniscectomy syndrome and K–L 0–3 degeneration treated with MAT reported that in a mean follow-up of 3.3 years, significant improvements were seen in most outcome measures, and 77% of patients returned to their desired level of play after MAT [6]. According to these results, it may be assumed that MAT is also beneficial to young athletic patients with moderate cartilage degeneration.

The reoperation rate was higher among patients with moderate K–L 2 knee OA (31%) than in patients with minimal K–L 0–1 OA (11%), with an overall reoperation rate of 20%. Similar results have been reported in previous studies [4, 16, 22, 24, 30], which might indicate that a higher degree of knee OA in terms of K–L classification and ICRS grade increases the incidence of degenerative tears of MATs.

This study has several limitations. First, the sample size was limited in this one-centre prospective follow-up study. Second, it was not possible to obtain data from all patients at all follow-up timepoints. In addition, as there was not a control group for comparison, what would have been the natural course of the patients´ disease without surgery can only be speculated. It can be assumed that MAT patients´ clinical outcomes after surgery were at a higher level, as full-thickness cartilage defects and knee osteoarthritis are irreversible and often progressive. MAT combined with corrective osteotomy or ACL reconstruction was performed for 37% of the patients, which make the patient material more heterogenous, and the obtained results should be evaluated with a certain caution.

The main strengths of our study were the systematic prospective follow-up protocol, including PROMs at 6 and 12 months and thereafter once a year, and the high follow-up percentage.

The results of the current study support for the treatment of young symptomatic patients after meniscectomy and failed further nonoperative management with MAT as it seems to result in pain relief and improved subjective satisfaction and function both in minimal and moderate knee OA. However, more data are required to evaluate the effect of concomitant procedures on the outcomes of MAT.

## Conclusion

MAT seems to increase patient satisfaction and patient outcome measures in patients with both K–L 0–1 and K–L 2 knee OA. No difference emerged in MAT survival at the 2-year follow-up between patients with low and moderate degrees of knee OA. The reoperation rate was higher among patients with moderate knee OA, but both groups benefited from MAT clinically. Knee OA progression in terms of K–L grade worsening was not observed in any patients.

## Data Availability

Data available on reasonable request from authors.

## References

[1] Beaufils P, Pujol N (2017). Management of traumatic meniscal tear and degenerative meniscal lesions. Save the meniscus. Orthop Traumatol Surg Res.

[2] Berliner JL, Brodke DJ, Chan V, SooHoo NF, Bozic KJ (2017). Can preoperative patient-reported outcome measures be used to predict meaningful improvement in function after TKA?. Clin Orthop Relat Res.

[3] Birchard Z, Herron T, Tuck JA (2021). Discoid meniscus.

[4] Bloch B, Asplin L, Smith N, Thompson P, Spalding T (2019). Higher survivorship following meniscal allograft transplantation in less worn knees justifies earlier referral for symptomatic patients: experience from 240 patients. Knee Surg Sports Traumatol Arthrosc.

[5] Cavendish P, DiBartola AC, Everhart JS, Kuzma S, Kim WJ, Flanigan DC (2020). Meniscal allograft transplantation: a review of indications, techniques, and outcomes. Knee Surg Sports Traumatol Arthrosc.

[6] Chalmers PN, Karas V, Sherman SL, Cole BJ (2013). Return to high-level sport after meniscal allograft transplantation. Arthroscopy.

[7] ElAttar M, Dhollander A, Verdonk R, Almqvist KF, Verdonk P (2011). Twenty-six years of meniscal allograft transplantation: is it still experimental? A meta-analysis of 44 trials. Knee Surg Sports Traumatol Arthrosc.

[8] Feeley BT, Lau BC (2018). Biomechanics and clinical outcomes of partial meniscectomy. J Am Acad Orthop Surg.

[9] Frobell RB, Roos EM, Roos HP, Ranstam J, Lohmander LS (2010). A randomized trial of treatment for acute anterior cruciate ligament tears. N Engl J Med.

[10] Gelber PE, Verdonk P, Getgood AM, Monllau JC (2017). Meniscal transplantation: state of the art. J ISAKOS.

[11] Grassi A, Di Paolo S, Coco V, Romandini I, Filardo G, Lucidi GA, Marcacci M, Zaffagnini S (2023). Survivorship and reoperation of 324 consecutive isolated or combined arthroscopic meniscal allograft transplants using soft tissue fixation. Am J Sports Med.

[12] Ha JK, Shim JC, Kim DW, Lee YS, Ra HJ, Kim JG (2010). Relationship between meniscal extrusion and various clinical findings after meniscus allograft transplantation. Am J Sports Med.

[13] Ha JK, Sung JH, Shim JC, Seo JG, Kim JG (2011). Medial meniscus allograft transplantation using a modified bone plug technique: clinical, radiologic, and arthroscopic results. Arthroscopy.

[14] Harris JD, Cavo M, Brophy R, Siston R, Flanigan D (2011). Biological knee reconstruction: a systematic review of combined meniscal allograft transplantation and cartilage repair or restoration. Arthroscopy.

[15] Jeong HW, Kim JS, Nam HS, Noh GS, Lee YS (2022). Assessment of anatomic restoration and clinical outcomes between medial and lateral meniscal allograft transplantation. Orthop J Sports Med.

[16] Kempshall PJ, Parkinson B, Thomas M, Robb C, Standell H, Getgood A, Spalding T (2015). Outcome of meniscal allograft transplantation related to articular cartilage status: advanced chondral damage should not be a contraindication. Knee Surg Sports Traumatol Arthrosc.

[17] Kunze KN, Davie RA, Ramkumar PN, Chahla J, Nwachukwu BU, Williams RJ (2023). Risk factors for graft failure after meniscal allograft transplantation: a systematic review and meta-analysis. Orthop J Sports Med.

[18] Lee BS, Kim HJ, Lee CR, Il BS, Lee DH, Kim NJ, Kim CW (2018). Clinical outcomes of meniscal allograft transplantation with or without other procedures: a systematic review and meta-analysis. Am J Sports Med.

[19] Lee HY, Il BS, Kim JM, Lee BS, Kim SM, Lee SJ (2023). Lateral meniscal allograft transplantation provides a chondroprotective effect on articular cartilage: quantitative 3-T magnetic resonance imaging T2 mapping. Arthroscopy.

[20] Lee S, Brown JR, Bartolomei C, Turnbull T, Miles JW, Dornan GJ, Frank RM, Vidal AF (2023). Effects of lateral opening-wedge distal femoral osteotomy on meniscal allograft transplantation A biomechanical evaluation. Orthop J Sports Med.

[21] Lubowitz JH, Verdonk PCM, Reid JB, Verdonk R (2007). Meniscus allograft transplantation: a current concepts review. Knee Surg Sports Traumatol Arthrosc.

[22] Mahmoud A, Young J, Bullock-Saxton J, Myers P (2018). Meniscal allograft transplantation: the effect of cartilage status on survivorship and clinical outcome. Arthroscopy.

[23] Mainil-Varlet P, Aigner T, Brittberg M, Bullough P, Hollander A, Hunziker E, Kandel R, Nehrer S, Pritzker K, Roberts S, Stauffer E (2003). Histological assessment of cartilage repair: a report by the Histology Endpoint Committee of the International Cartilage Repair Society (ICRS). J Bone Jt Surg Am.

[24] McCormick F, Harris JD, Abrams GD, Hussey KE, Wilson H, Frank R, Gupta AK, Bach BR, Cole BJ (2014). Survival and reoperation rates after meniscal allograft transplantation: analysis of failures for 172 consecutive transplants at a minimum 2-year follow-up. Am J Sports Med.

[25] Ogura T, Ackermann J, BarbieriMestriner A, Merkely G, Gomoll AH (2020). Minimal clinically important differences and substantial clinical benefit in patient-reported outcome measures after autologous chondrocyte implantation. Cartilage.

[26] Sihvonen R, Paavola M, Malmivaara A, Itälä A, Joukainen A, Kalske J, Nurmi H, Kumm J, Sillanpaä N, Kiekara T, Turkiewicz A, Toivonen P, Englund M, Taimela S, Järvinen T (2020). Arthroscopic partial meniscectomy for a degenerative meniscus tear: a 5 year follow-up of the placebo-controlled FIDELITY (Finnish Degenerative Meniscus Lesion Study) trial. Br J Sports Med.

[27] Smith NA, Costa ML, Spalding T (2015). Meniscal allograft transplantation: rationale for treatment. Bone Jt J.

[28] Smith NA, MacKay N, Costa M, Spalding T (2015). Meniscal allograft transplantation in a symptomatic meniscal deficient knee: a systematic review. Knee Surg Sports Traumatol Arthrosc.

[29] Smith NA, Parsons N, Wright D, Hutchinson C, Metcalfe A, Thompson P, Costa ML, Spalding T (2018). A pilot randomized trial of meniscal allograft transplantation versus personalized physiotherapy for patients with a symptomatic meniscal deficient knee compartment. Bone Jt J.

[30] Stone KR, Pelsis JR, Surrette ST, Walgenbach AW, Turek TJ (2015). Meniscus transplantation in an active population with moderate to severe cartilage damage. Knee Surg Sports Traumatol Arthrosc.

[31] Vasta S, Zampogna B, Den HT, El Bitar Y, Uribe-Echevarria B, Amendola A (2022). Outcomes, complications, and reoperations after meniscal allograft transplantation. Orthop J Sports Med.

[32] Verdonk PCM, Verstraete KL, Almqvist KF, De Cuyper K, Veys EM, Verbruggen G, Verdonk R (2006). Meniscal allograft transplantation: long-term clinical results with radiological and magnetic resonance imaging correlations. Knee Surg Sports Traumatol Arthrosc.

[33] Vundelinckx B, Bellemans J, Vanlauwe J (2010). Arthroscopically assisted meniscal allograft transplantation in the knee: a medium-term subjective, clinical, and radiographical outcome evaluation. Am J Sports Med.

[34] Wagner KR, Kaiser JT, Knapik DM, Condron NB, Gilat R, Meeker ZD, Sivasundaram L, Yanke AB, Cole BJ (2023). Patient-specific variables associated with failure to achieve clinically significant outcomes after meniscal allograft transplantation at minimum five years’ follow-up. Arthroscopy.

[35] Wagner KR, Kaiser JT, Hevesi M, Cotter EJ, Gilat R, Meeker ZD, Frazier LP, Yanke AB, Cole BJ (2023). Minimum 10-year clinical outcomes and survivorship of meniscal allograft transplantation with fresh-frozen allografts using the bridge-in-slot technique. Am J Sports Med.

[36] van der Wal RJP, Nieuwenhuijse MJ, Spek RWA, Thomassen BJW, van Arkel ERA, Nelissen RGHH (2020). Meniscal allograft transplantation in The Netherlands: long-term survival, patient-reported outcomes, and their association with preoperative complaints and interventions. Knee Surg Sports Traumatol Arthrosc.

[37] Yoon KH, Lee SH, Park SY, Kim HJ, Chung KY (2014). Meniscus allograft transplantation: a comparison of medial and lateral procedures. Am J Sports Med.

[38] Yow BG, Donohue M, Tennent DJ (2021). Meniscal allograft transplantation. Sports Med Arthrosc Rev.

[39] Zaffagnini S, Grassi A, MarcheggianiMuccioli GM, Benzi A, Serra M, Rotini M, Bragonzoni L, Marcacci M (2016). Survivorship and clinical outcomes of 147 consecutive isolated or combined arthroscopic bone plug free meniscal allograft transplantation. Knee Surg Sports Traumatol Arthrosc.

[40] Zaffagnini S, Romandini I, Filardo G, Dal Fabbro G, Grassi A (2023). Meniscal allograft transplantation, anterior cruciate ligament reconstruction, and valgus high tibial osteotomy for meniscal-deficient, unstable, and varus knees: surgical technique and clinical outcomes. Int Orthop.

